# Detection of Differentially Expressed MicroRNAs in Rheumatic Heart Disease: miR-1183 and miR-1299 as Potential Diagnostic Biomarkers

**DOI:** 10.1155/2015/524519

**Published:** 2015-10-11

**Authors:** Ni Li, Jiangfang Lian, Sheng Zhao, Dawei Zheng, Xi Yang, Xiaoyan Huang, Xinbao Shi, Lebo Sun, Qingyun Zhou, Huoshun Shi, Guodong Xu, Enchill KoJo Incoom, Jianqing Zhou, Guofeng Shao

**Affiliations:** ^1^Ningbo Medical Center Lihuili Hospital, Ningbo, Zhejiang 315041, China; ^2^Yinzhou Second Hospital, Ningbo, Zhejiang 315040, China; ^3^Ningbo University School of Medicine, Ningbo, Zhejiang 315211, China

## Abstract

This study compared microRNA (miRNA) expression profiles between rheumatic heart disease (RHD) patients and healthy controls to investigate their differential expression and help elucidate their mechanisms of action. Microarray analysis was used to measure miRNA expression, and a total of 133 miRNAs were shown to be significantly upregulated in RHD patients compared with controls, including miR-1183 and miR-1299. A total of 137 miRNAs, including miR-4423-3p and miR-218-1-3p, were significantly downregulated in RHD patients. Quantitative real-time-PCR confirmed microarray findings for miR-1183 and miR-1299 in both tissue and plasma. Bioinformatic predictions were also made of differentially expressed miRNAs as biomarkers in RHD by databases and GO/pathway analysis. Furthermore, we investigated miR-1183 and miR-1299 expression in RHD patients with secondary pulmonary hypertension (PAH). Our findings identified an important role for miR-1299 as a direct regulator of RHD, while the observed difference in expression of miR-1183 between RHD-PAH patients with high or low pulmonary artery pressure suggests that miR-1183 overexpression may reflect pulmonary artery remodeling. miR-1183 and miR-1299 appear to play distinct roles in RHD pathogenesis accompanied by secondary PAH and could be used as potential biological markers for disease development.

## 1. Introduction

MicroRNAs (miRNAs) are a class of single-stranded endogenous noncoding RNA molecules [1, 2], approximately 22 nucleotides (nt) in length, that negatively regulate gene expression by targeting the 3′-untranslated region of specific mRNAs for degradation or translational repression [3, 4]. The rapid growth in miRNA studies has demonstrated that they play an important role in a range of biological processes and are viewed as critical regulators in immune cell lineage commitment, differentiation, maturation, and immune signaling pathways [5, 6]. Additionally, deregulated miRNA expression patterns have been documented in many human diseases including inflammatory and autoimmune diseases [7–9].

Early miRNA studies focused on their role in cancer [10–13]; however, more recently, there has been a shift of attention to their possible impact on cardiovascular development and diseases [14]. Indeed, they are important in tissue development and influence the pathological processes of many cardiovascular diseases, including acute myocardial infarction, heart failure, coronary artery disease, stroke, and hypertension [1, 2]. To the best of our knowledge, the effects of miRNAs on the development of rheumatic heart disease (RHD) in either healthy tissues or circulating plasma have not yet been investigated.

RHD is primarily an autoimmune sequela of an acute rheumatic fever [15, 16], which occurs as a result of beta-hemolytic streptococcal infection [17, 18]. RHD can cause chronic inflammation of the endocardium and myocardium, leading to valvular dysfunction and hemodynamic changes [19] and, commonly, heart failure, stroke, or other serious related complications. Unfortunately, because of the lack of a specific method of detecting RHD, many patients have been diagnosed with irreversible valvular dysfunction, for which valvular surgery is one of the main treatments. RHD continues to be a burden in several developing countries such as India and China, though it is reasonably rare in western countries—probably because of the widespread use of antibiotics [20–22]. Therefore, the identification of a biomarker of characteristic RHD pathophysiology will be valuable to aid early detection and enable patients to avoid surgery by starting effective treatment at an early stage.

Pulmonary arterial hypertension (PAH) is a common complication of many RHD patients. Because PAH is characterized by the enhanced proliferation and reduced apoptosis of pulmonary artery smooth muscle cells [23], and as some miRNAs are also associated with the regulation of cell proliferation and apoptosis, it is hypothesized that they might be implicated in the etiology of PAH [22–26].

Therefore, in this study, we analyzed the miRNA expression profiles of RHD patients using microarray and confirmed our findings using quantitative real-time- (qRT-) PCR. We screened the roles of differentially expressed miRNAs in RHD with secondary PAH and used bioinformatics to predict and analyze their target genes as potential biomarkers of RHD. We also propose new directions for their potential therapeutic use in RHD.

## 2. Materials and Methods

### 2.1. Patients

A total of 100 subjects were selected for the study from the Inpatient Clinic of Ningbo Medical Center, Lihuili Hospital (Ningbo, China), between March 2012 and October 2013. Of these subjects, 50 were RHD patients (case group), and the remaining 50 were normal healthy adults (control group) with no medical history of congenital heart disease, cardiomyopathy, or liver or renal diseases. The inclusion criteria of the RHD group are as follows: (i) every patient diagnosed with mitral valve prolapse because of mitral chordae tendineae fracture and mitral insufficiency and scheduled for mitral valve replacement; (ii) left ventricular ejection fraction (EF) > 50%; (iii) left ventricular end-diastolic diameter (LVEDD) < 55 mm. RHD cases and their controls were well matched based on the following details: (iv) same gender; (v) difference of age < 5 years; (vi) other physiological indexes from physical check in close. All human materials were obtained in accordance with the hospital's regulations and hence were approved by the Ethics Committee of Lihuili Hospital. Written informed consent was also obtained from all subjects in advance.

### 2.2. Sample Collection

Blood samples were collected in EDTA tubes for plasma collection from the 50 RHD cases and stored at −80°C. All blood samples of cases and controls were collected by the same investigators. Left ventricular papillary muscles were obtained from 12 cases of resected mitral valves from RHD cases. They were later transferred into a physiological saline solution and then into liquid nitrogen and stored at −80°C. Six normal tissues for comparison were obtained from donors who had died from trauma.

### 2.3. RNA Isolation and Characterization

Peripheral blood was coagulated at room temperature for 30 min then centrifuged at 3000 rpm for 15 min to completely remove cell debris. It was stored at −80°C until required for miRNA detection. Total RNA was extracted from 625 *μ*L of plasma using the mirVana PARIS kit (Ambion, USA) according to the manufacturer's instructions. Total RNA was also extracted from 10–50 mg left ventricular papillary muscles using the mirVana isolation kit according to the manufacturer's protocol (Ambion) [27–29]. The final elution volume of all RNA samples was 100 *μ*L, and concentrations were determined by the ultramicro nucleic acid ultraviolet tester (NANODROP 1000, Wilmington, USA). RNA was reverse-transcribed into cDNA using the TaqMan microRNA reverse transcription kit (Applied Biosystems, Foster City, CA) using miRNA-specific primers provided by the manufacturer in Applied BioSystems 9700 Thermocycler. All cDNAs were stored at −20°C.

### 2.4. Quantitative Real-Time-PCR

Quantitative real-time-PCR (qRT-PCR) was performed as previously described [30]. Each reaction was performed in a final volume of 10 *μ*L containing 4.5 *μ*L cDNA, 5 *μ*L TaqMan Universal PCR Master Mix (No AmpErase), and 0.5 *μ*L TaqMan miRNA Assay (Applied Biosystems). The thermal cycle was set as start with 10 min template denaturation at 95°C, 40 cycles of denaturation at 95°C for 15 s, and combined primer annealing/elongation at 60°C for 1 min. Each sample was run in triplicate, and noncoding small RNA RNU6B was used as the internal control gene, according to the Applied Biosystems Application Note. RNU6B has previously been demonstrated to have both abundant and stable expression across 38 different human tissues and organs. It is regarded as one of the control genes with the least variability for miRNA assays and has been widely used in different fields, including cardiovascular research. We used Taqman microRNA assay for the qRT-PCR. The primer sequences were searched on the ABI official website, shown as follows.

The assay ID of miR-1183 assay is 002841; hsa-miR-1183 mature miRNA sequence is CACUGUAGGUGAUGGUGAGAGUGGGCA. The assay ID of miR-1299 assay is 241065_mat; hsa-miR-1299 mature miRNA sequence is UUCUGGAAUUCUGUGUGAGGGA. The assay ID of RNU6B assay is 001093; control RNU6b sequence is CGCAAGGATGACACGCAAATTCGTGAAGCGTTCCATATTTTT.

### 2.5. miRNA Microarray Analysis

To study the differential expression of miRNAs in RHD patients, we performed miRNA expression profiling on plasma samples using the miRCURY LNA Array system because of its comprehensive profiling and high sensitivity and specificity (see S2_Fig in Supplementary Material available online at http://dx.doi.org/10.1155/2015/524519). Here we selected 3 patients named numbers 1, 2, and N1-3 for microarray assay from the above 50 RHD patients. Meanwhile, we also selected 3 healthy controls named numbers 3, 4, and 5 from 50 control groups, which are also evaluated for microarray assay. Microarray analysis was performed by Kangcheng Bio-tech Inc. (Shanghai, China). miRNAs selected for investigation in our study were further filtered on the basis of expression levels and previously published data [31]. The RNA of each individual was analyzed on a separate chip. Data were analyzed in Genepix Pro 6.0 and saved as EXCEL files. GeneSpring 7.2 was used for further data analysis. Fold changes in miRNAs between groups, either twofold greater or less, were considered to represent differential expression.

### 2.6. Statistical Analysis

Experimental data were analyzed using SPSS19.0 statistical software. RNA concentration and Ct value levels were presented as means ± SD.

## 3. Results 

### 3.1. RNA Concentration of RHD Patients in Plasma and Tissue

First, we validated the feasibility of miRNA detection from plasma in all subjects. No differences in RNA concentrations were observed among the different groups (plasma RHD group: 17.74 ± 5.59 ng/*μ*L; plasma control group: 16.43 ± 4.32 ng/*μ*L; tissue RHD group: 40.95 ± 27.90 ng/*μ*L; tissue control group: 52.28 ± 28.21 ng/*μ*L) (S1_Fig). However, the lower RNA yield from plasma hampered the actual RNA quantification compared with that from tissue (S1_Fig).

### 3.2. Genome-Wide Expression Profiling of Plasma miRNAs by Microarray Analysis

**Figure 1:**
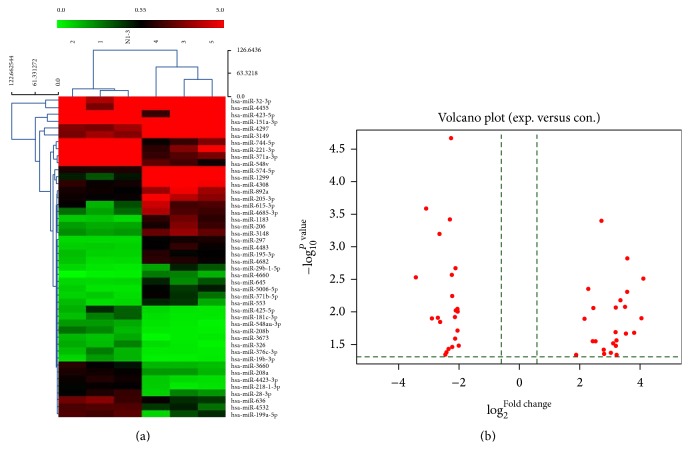
MicroRNA microarray expression data from plasma samples of rheumatic heart disease (*n* = 3) and healthy control subjects (*n* = 3). (a) Heat map of microRNA microarray expression data from plasma samples of rheumatic heart disease (*n* = 3) and healthy control subjects (*n* = 3). The expression of miRNA is hierarchically clustered on the *y*-axis, and RHD plasma samples or healthy control plasma samples or healthy control plasma samples are hierarchically clustered on the *x*-axis. The legend on the right indicates the miRNA represented in the corresponding row. The relative miRNA expression is depicted according to the color scale shown on the right. Red indicated upregulation; blue indicated downregulation; numbers 1, 2, and N1-3 indicate healthy control samples; numbers 3, 4, and 5 indicate RHD plasma samples. (b) Volcano plot from gene chips represents different fold changes from many upregulated miRNAs and downregulated miRNAs.

Hundreds of miRNAs showed differential expression between RHD cases and controls (S2_Fig). A total of 133 miRNAs, including miR-1299 and miR-1183, were significantly upregulated (>2-fold), while 137 were significantly downregulated (>2-fold), including miR-4423-3p and miR-218-1-3p (S3_Fig). We selected several significantly differentially expressed miRNAs for further study. Gene chips represents different fold changes from many upregulated miRNAs and downregulated miRNAs, either by heat map of microRNA microarray expression data (Figure 1(a)) or volcano plot (Figure 1(b)). Chip results suggested that miR-1299 and miR-1183 expressed in RHD were significantly upregulated (>10-fold), whereas miR-4423-3p and miR-218-1-3p expressed in RHD were significantly downregulated (>5-fold) in RHD cases compared with controls (Table 1).

### 3.3. Plasma and Tissue miR-1183 and miR-1299 Expressions Are Potential Biochemical Marker for RHD by Quantitative Real-Time-PCR

**Figure 2:**
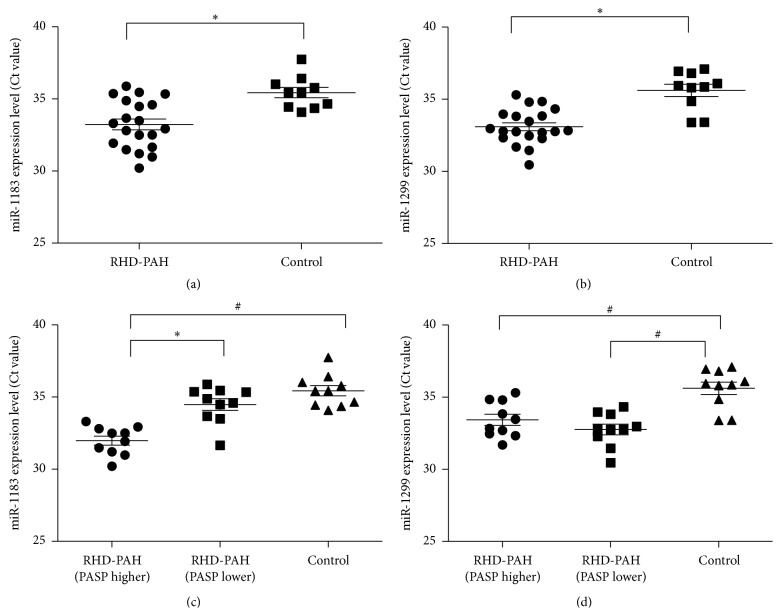
Plasma level of representative differentially expressed miR-1183 and miR-1299 in 20 RHD patients and 10 normal controls (^*∗*^ *P* < 0.05, ^#^ *P* < 0.01).

**Figure 3:**
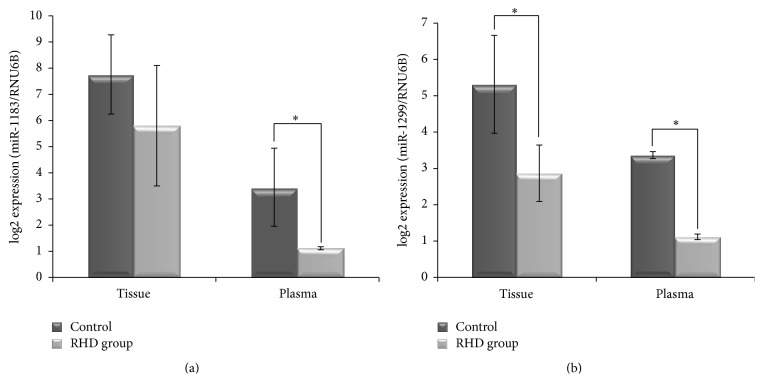
Upregulation of microRNA-1183 and microRNA-1299 expression in rheumatic heart disease tissues and plasma by real-time RT-PCR. (a) The differential expression of miRNA-1183 in the tissue and plasma of RHD patients and normal controls. (b) The differential expression of miRNA-1299 in the tissue and plasma of RHD patients and normal controls (^*∗*^ *P* < 0.05).

To verify the accuracy of the microarray-based miRNA measurements, expression levels of hsa-miR-1183, hsa-miR-1299, and RNU6B were assessed using qRT-PCR [32]. All miRNAs including RNU6B showed reliable Ct values in most samples (Figure 2), and fluorescent signals failed to reach the set threshold after 40 cycles in very few assays. Results are shown in Figures 2 and 3; qRT-PCR revealed significant differences in the expression of miRNA-1183, miRNA-1299, and RNU6B in both tissue and plasma samples of RHD patients compared with healthy controls, which was consistent with microarray data. Ct values in RHD tissue for miR-1183 were 34.62 ± 2.23, 31.81 ± 2.46 for miR-1299, and 28.33 ± 3.48 for RNU6B.

Pulmonary artery systolic pressure (PASP) is a key factor to evaluate the severity of PAH, so we next divided RHD patients with secondary PAH into two groups: those with high PASP (RHD-PAH [PASP higher >40 mmHg]) and those with low PASP (RHD-PAH [PASP lower <40 mmHg]) (Table 2). The expression of miR-1183 was significantly upregulated in both RHD plasma samples (Figure 2(a), *P* = 0.012) and the subset of RHD cases with high PASP (Figure 2(b), *P* = 0.021). By contrast, although the expression of miR-1299 was significantly upregulated in RHD plasma samples (Figure 2(c), *P* = 0.011), no significant difference was observed between the subsets with high and low PASP (Figure 2(d), *P* = 0.566). Moreover, the overexpression of miR-1183 in high versus low PASP cases could reflect the pulmonary artery remodeling of PAH secondary to RHD, meaning that it plays a more important role in secondary PAH complications than primary disease. Although the same trend was also observed when comparing miRNA-1183 in tissue samples between the two groups by qRT-PCR (Figure 3(a)), the difference was not significant. Interestingly, the expression of miRNA-1299 is significantly higher in the RHD group compared with the non-RHD group for both tissue and plasma (Figure 3(b), *P* < 0.05).

### 3.4. Bioinformatic Analysis and Predicted miRNA Molecular Targets Identified in RHD Patients

**Figure 4:**
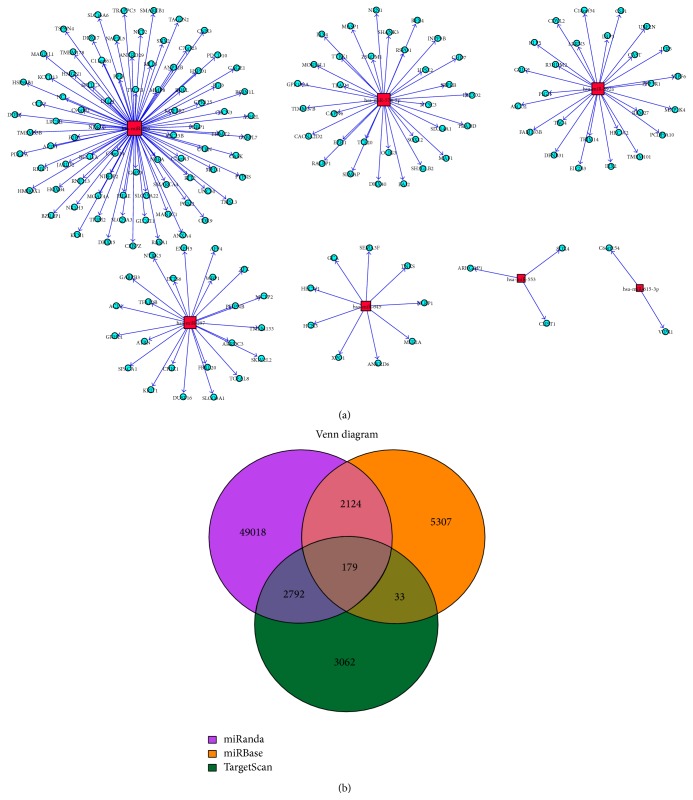
Target genes of differential miRNA expression predicted. (a) MicroRNA–mRNA-Gene-Network of several representative miRNAs with their predicted target genes. (b) Overlapping data of three databases from the target summary by miRBase (http://www.mirbase.org/), miRanda (http://www.microrna.org/), and TargetScan (http://www.targetscan.org/).

Those miRNAs showing significantly differential expression by microarray were analyzed by bioinformatics, including target gene prediction, gene ontology (GO) analysis, and pathway analysis, with the aim of investigating target genes and regulatory mechanisms. Target genes of differential miRNA expression were predicted using three algorithms: miRBase (http://www.mirbase.org/), miRanda (http://www.microrna.org/), and TargetScan (http://www.targetscan.org/) (Figure 4). Only genes identified by all three algorithms were considered to be the predicted targets for each miRNA.

**Figure 5:**
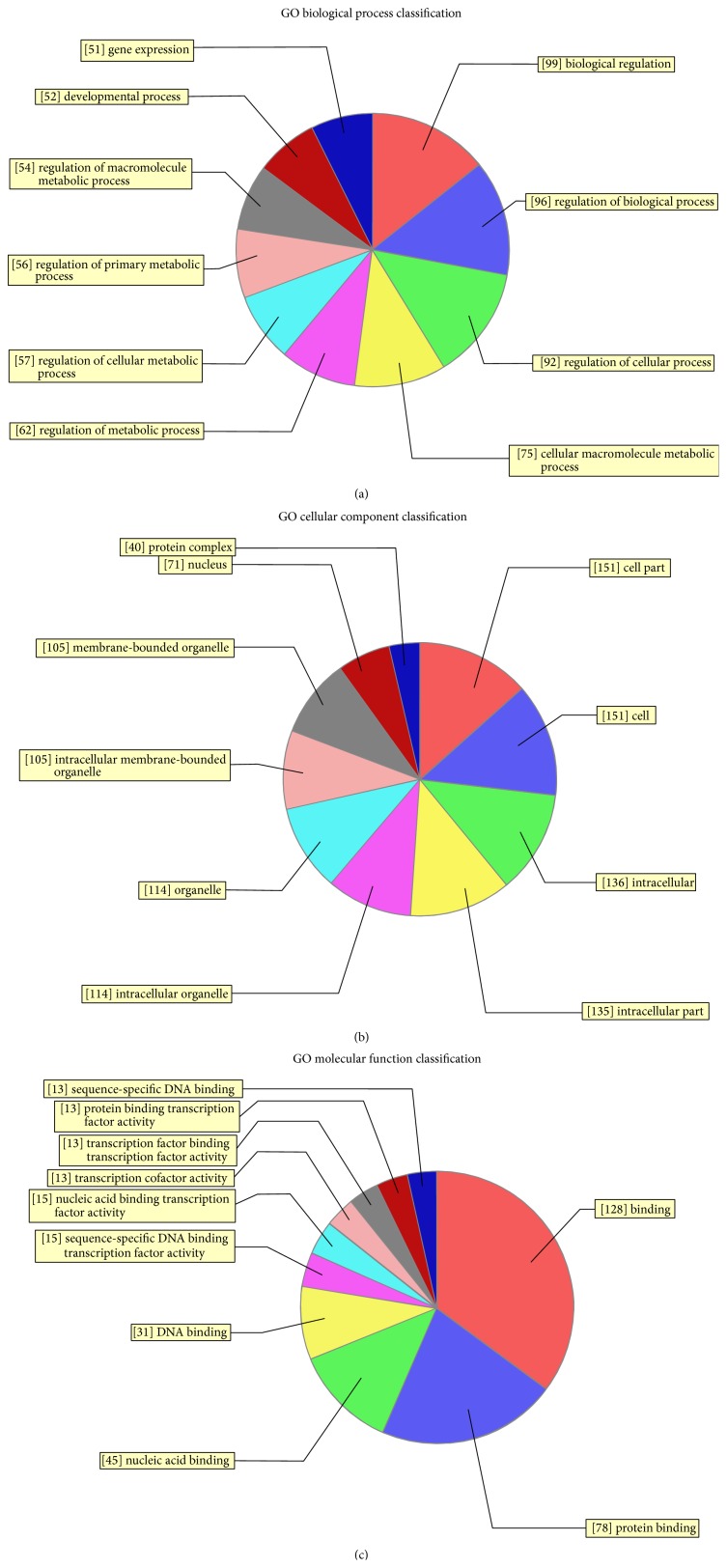
GO analysis classifications of predicted target genes regulated by differentially expressed microRNAs (miRNAs) in rheumatic heart disease. GO analysis was performed on genes predicted to be targets of differentially expressed miRNAs.

**Figure 6:**
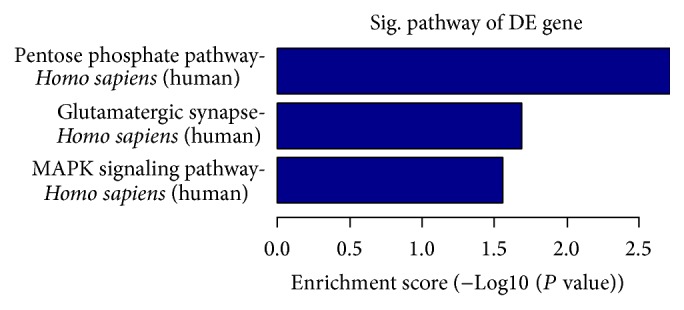
Pathway classifications of predicted target genes regulated by differentially expressed microRNAs (miRNAs) in rheumatic heart disease. Pathway analysis was performed on genes predicted to be targets of differentially expressed miRNAs. The negative log of the *P* value (log⁡⁡10  *P*) is plotted on the *x*-axis.

Targets were found to be predominantly involved in the regulation of cellular and biological processes, including SOX family members and* MEF2A*. Hundreds of target genes were predicted and were mainly involved in transcription coactivator activity, RNA polymerase II transcription coactivator activity, histone-lysine N-methyl transferase activity, protein-lysine N-methyl transferase activity (GO molecular function), the positive regulation of biological processes, histone-lysine methylation, protein alkylation (GO biology process), and intracellular roles (GO cellular component) (Figure 5, S4_Fig). To assess the possible biological impact of the differentially expressed miRNAs, we undertook pathway analyses of the predicted target genes, revealing that the gene set was mostly involved in biological pathways including the pentose phosphate pathway, glutamatergic synapse, and the MAPK signaling pathway (Figure 6).

Because of the differences in miR-1183 and miR-1299 expression, we separately predicted the target genes for these two miRNAs.* Bcl-2* was predicted to be an important target gene of miR-1299, which may influence cardiomyocyte apoptosis. Considering RHD as an autoimmune sequela of an acute rheumatic fever, it can cause chronic inflammation. PBMC and THP-1 cells as classical immune cells are studied to dig deep into the mechanism study including both miR-1183 and miR-1299 mimic and inhibitor study. Series of studies are in progress including identifying the changes of target genes Bcl-2 and EGFR mRNA expression and related cytokines (data not shown). Up to now, we have already further studied the relationship of miR-1299 mimic, inhibitor, and Bcl-2 expression levels. It seems that miR-1299 mimics can upregulate miRNA level and induce cardiomyocyte apoptosis (data not shown). Meanwhile, CXCR4, EGF, and EGFR were also predicted to be important target genes of miR-1183. However, the following mechanism study including miR mimic was still in progress.

## 4. Discussion

Several studies have shown that a variety of miRNAs are implicated in cardiovascular diseases, so exploratory research has been conducted into the possibility of using them as biological markers based on their expression in plasma or tissue [33–35]. However, this has not been tested in RHD. The present miRNA array results suggested that miR-1299, miR-1183, and so forth are significantly upregulated, while miR-4423-3p, miR-218-1-3p, and so forth are significantly downregulated in RHD. qRT-PCR confirmed that the expression of miR-1299 and miR-1183 in RHD tissue and plasma was significantly higher in RHD cases than healthy controls. Moreover, the enhanced levels of miR-1183 and miR-1299 expression in plasma are consistent with those in tissues, suggesting that they could be used as potential biological markers in RHD. Besides, some other miRNAs determined to be expressed differently in RHD compared to healthy controls can be dug deeper, like miR206, miR208a, miR208b, and miR574-5p which have already been studied in the regions of other cardiovascular diseases [36–38]. To explore the possible functions on RHD of these miRNAs may provide us ideas whether they are potent therapeutic targets for cardiac hypertrophy, fibrosis, dysfunction, and so forth.

Pulmonary arterial hypertension is a debilitating condition with progressive remodeling of the pulmonary resistance vessels [22]. It is characterized by excessive vascular resistance and smooth muscle cell proliferation in small pulmonary arteries and finally causes elevation of pulmonary vascular resistance, right ventricular failure, and death [39, 40]. Variations in PASP in RHD patients are likely to influence the expression of different miRNAs in RHD-PAH.

We identified an important role for miR-1299 as a direct regulator of RHD. Meanwhile, the difference in expression of miR-1183 between RHD cases with high and low PASP suggests that its overexpression is caused by the pulmonary artery remodeling of PAH secondary to RHD, meaning that it plays a more important role in secondary complications than primary disease. miR-1183 and miR-1299 may therefore have an independent effect on the disease processes of RHD and PAH. We searched the literature for mechanistic articles on miR-1183 or miR-1299 and found them to be extremely limited. miR-1183 was previously reported to be downregulated in tick salivary glands [41], while another study reported that it has a relationship with a functional polymorphism in the EpCAM gene [42]. miR-1183 is also known to be upregulated in locally advanced rectal cancer patients [43]. Information about miR-1299 is even more limited, so further detailed studies are required for a greater understanding of the molecular mechanisms of miR-1183 and miR-1299.

Based on the work which we have done for screening differentially expressed miRNAs (e.g., miR-1183 and miR-1299) of RHD, most importantly, evaluation of the stability and effect of miRNA-based therapeutics is of great importance for the comprehensive understanding of the miR-1183 and miR-1299 functions in rheumatic heart disease. The bioinformatics analysis is often used to further study the mechanism of the differential expression of miRNAs [44–46] and to predict and analyze their target genes with the aim of understanding regulatory mechanisms in the future [47, 48]. Our analysis predicted hundreds of target genes, and pathway and GO analyses demonstrated that the gene set was mostly involved in biological pathways and cellular processes such as the pentose phosphate pathway, glutamatergic synapse, and MAPK signaling pathway. Our findings also suggest that the regulation of cellular and metabolic processes may influence the release of miR-1299 and miR-1183 into the peripheral blood following their overexpression in the tissue. The* Bcl-2* gene and its influence on cardiomyocyte apoptosis may be an important factor in association with miR-1299 expression. However, more detailed studies are needed for a greater understanding of this process.

## 5. Conclusion

Taken together, our data revealed the differential expression of specific miRNAs in RHD accompanied by secondary PAH. miR-1183 and miR-1299 appear to play a distinct role in disease pathology and so could be potential biological markers for both RHD and PAH. Future functional and mechanistic studies on the dynamic changes of miRNA expression in RHD may improve our understanding of the regulatory role of miRNAs in RHD.

## Supplementary Material

Genome-wide miRNA profiling using miChip.

## Figures and Tables

**Table 1:** List of the hsa-miRNAs with at least 2-fold changes.

Upregulated miRNA (>10 folds)			Downregulated miRNAs (>5 folds)		
miRNA	Folds	*P* value	miRNA	Folds	*P* value
miR-1299	17.13272	0.003132	miR-4423-3p	0.092672	0.002951
miR-1183	16.5075	0.012476	miR-218-1-3p	0.117514	0.000261
miR-4455	13.98099	0.020973	miR-744-5p	0.13412	0.01252
miR-3148	11.95398	0.001515	miR-4666a-5p	0.139837	0.018463
miR-4660	11.9266	0.004957	miR-208b	0.154499	0.012267
miR-3149	11.56086	0.021548	miR-199a-5p	0.159151	0.000643
miR-4682	11.37554	0.008373	miR-548v	0.162247	0.014474
miR-297	10.12601	0.006702	miR-3660	0.184096	0.045429
miR-206	9.313578	0.045881	miR-28-3p	0.188257	0.041912
miR-32-3p	9.312469	0.027754	miR-425-5p	0.196498	0.037533
miR-4308	9.191765	0.020532	miR-548-3p	0.203344	0.000383
miR-4483	9.155266	0.033305	miR-4532	0.209924	0.000217
miR-574-5p	9.132102	0.008565	miR-3673	0.211119	0.0027
miR-4685-3p	8.583969	0.03023	miR-221-3p	0.215457	0.005767
miR-4297	8.120556	0.042287	miR-181c-3p	0.216058	0.034691
miR-4722-5p	7.657944	0.002658	miR-636	0.226805	0.011922
miR-3591-5p	7.405006	0.020074	miR-376c-3p	0.227146	0.025902
miR-615-3p	7.082924	0.044276	miR-4798-3p	0.228792	0.038073
miR-195-3p	6.976838	0.039299	miR-208a	0.231132	0.002143
miR-4657	6.682702	0.00013	miR-371a-3p	0.233856	0.009664
miR-3657	6.671498	0.024803	miR-4708-5p	0.23388	0.010049
miR-5006-5p	6.639262	0.000398	miR-151a-3p	0.239967	0.00974
miR-4458	6.188047	4.05*E* − 06	miR-4678	0.240886	0.004481
miR-29b-1-5p	5.735279	0.028064	miR-423-5p	0.240937	0.009271
miR-205-3p	5.521096	0.008749	miR-326	0.243204	0.019519
miR-498	5.497149	0.017617	miR-4703-3p	0.244857	0.021779
miR-4450	5.491613	0.026159	miR-19b-3p	0.24777	0.033327
miR-371b-5p	5.378559	0.028034	miR-3612	0.250328	0.017766
miR-2113	5.371673	0.025333	miR-4748	0.252973	0.026891
miR-3689a-5p	5.342335	0.015381	miR-186-5p	0.253236	0.045289
miR-3120-5p	5.221742	0.00234	miR-320b	0.253705	0.029743
miR-4681	5.200976	0.01057	miR-550b-2-5p	0.253829	0.045176
miR-3690	5.145123	0.015582	miR-346	0.254841	0.021562
miR-4804-3p	5.03806	0.013518	miR-130a-3p	0.256733	0.04919
miR-4447	4.964015	0.001608	miR-4650-5p	0.256936	0.02673
miR-892a	4.895473	0.004484	miR-2682-5p	0.257933	0.008486
miR-4795-5p	4.698387	0.000309	miR-219-5p	0.263898	0.011573
miR-3158-3p	4.618894	0.003654	miR-337-3p	0.265439	0.016578

**Table 2:** Baseline characteristics of different severe pulmonary arterial hypertension complication on RHD patients and healthy control subjects.

Groups	Group 1: high pulmonary artery pressure group in RHD patients (*n* = 10)	Group 2: low pulmonary artery pressure group in RHD patients (*n* = 10)	Group 3: control (*n* = 10)
Age, years	54.2 ± 5.73	59.2 ± 6.27	49.6 ± 7.0
Sex, males/females	3/7	4/6	5/5
NYHA grades	II~III	IV	/
PASP (mmHg)	88.3 ± 5.376	33.1 ± 4.55	/

## Abbreviation

RHD: Rheumatic heart disease
miR: MicroRNAs
PAH: Pulmonary hypertension
QRT-PCR: Quantitative real-time-PCR
PASP: Pulmonary artery systolic pressure
nt: Nucleotides.
