# Supplementary material for: Detection of Differentially Expressed MicroRNAs in Rheumatic Heart Disease: miR-1183 and miR-1299 as Potential Diagnostic Biomarkers
Source: Biomed Res Int. 2015 Oct 11;2015:524519. doi: 10.1155/2015/524519 (PMC4619814; doi:10.1155/2015/524519)
Supplement: Supplementary file 1 — Genome-wide miRNA profiling using miChip. [file 524519.f1.pdf]

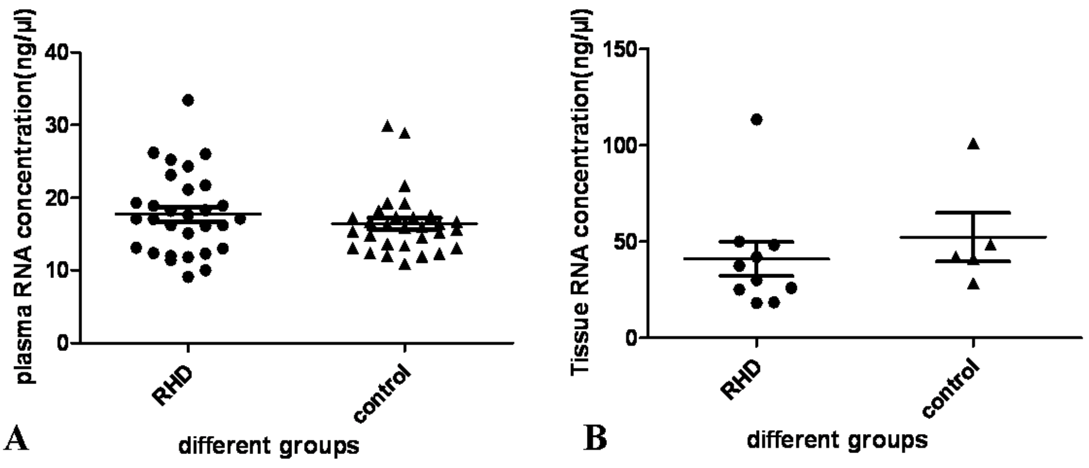

S1_Fig RNA Quantification and Quality Assurance by NanoDrop ND-1000(A: Plasma samples; B: Tissue samples)

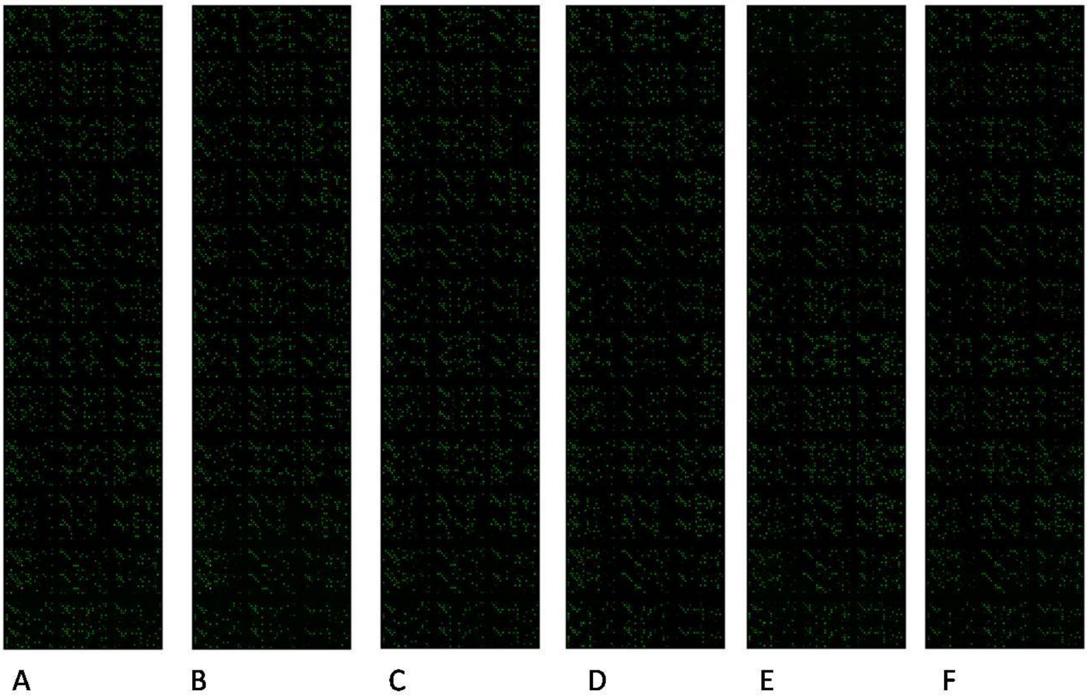

S2_Fig Detection of miRNAs by miRCURY™ LNAs microarrays

Total RNAs extracted from virgin pregnancy lactation and involution mouse mammary gland tissues were covalently labeled with Hy3 (green channel) and hybridized to the array. The microarray slides contained four replicate subarrays, control group(A、B、C), RHD group(D、E、F), respectively.

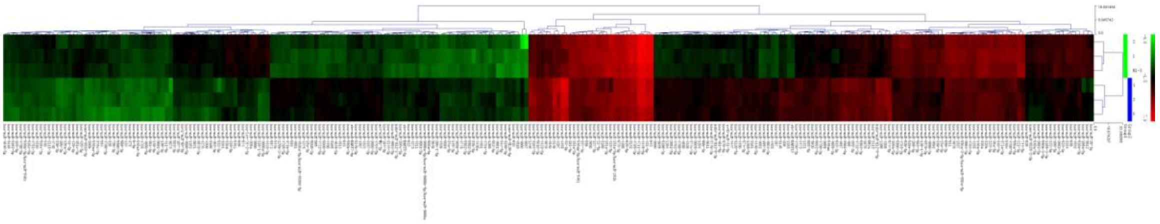

S3_Fig Genome-wide miRNA profiling using miChip. miRNA expression profiles were monitored across plasma samples of rheumatic heart disease(n=3) and healthy control subjects(n=3). Data were organized according to the expression levels of individual miRNA. The key color bar indicates miRNA expression levels (dark red indicates high expression, whereas, dark green indicates no detectable expression).

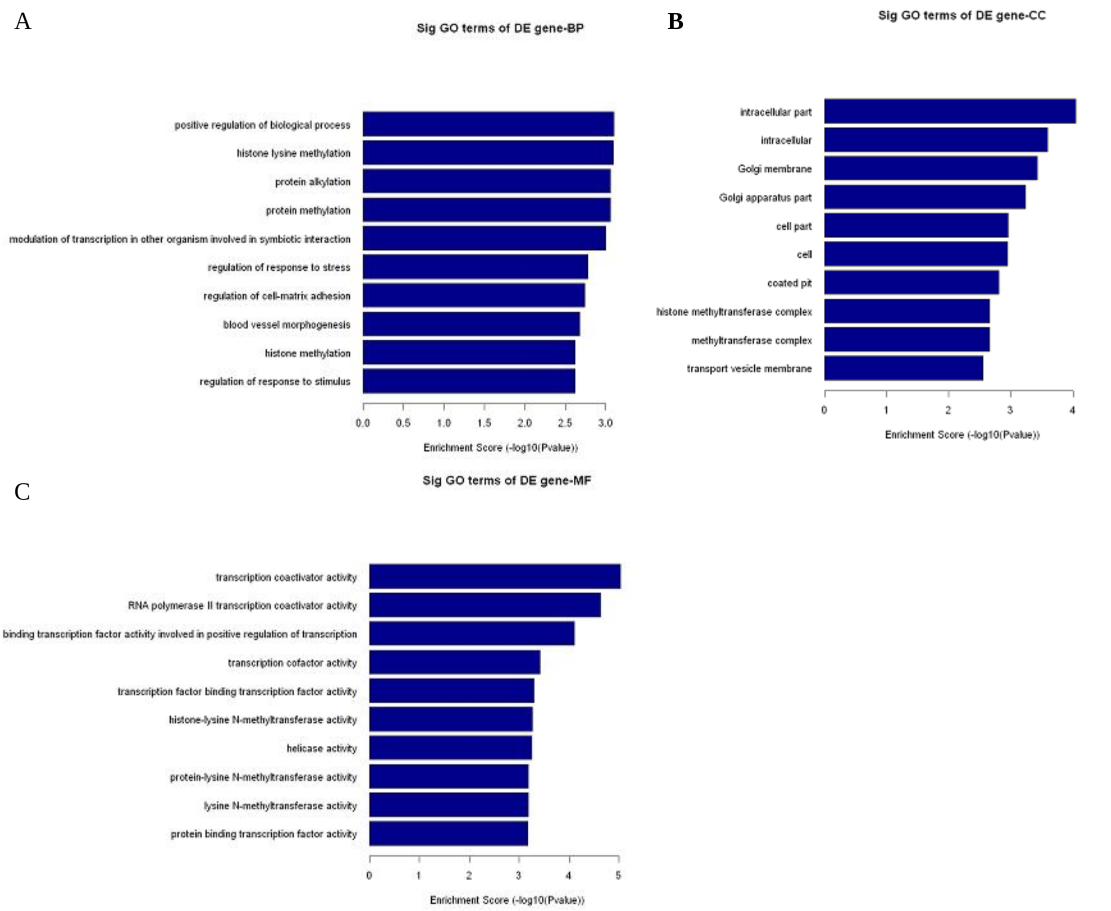

S4_Fig GO analysis was performed on genes predicted to be targets of differentially expressed miRNAs. The negative log of the P value (log10P) is plotted on the x-axis. The gene ontology covers three domains: Biological Process(A: BP), Cellular Component(B:CC) and Molecular Function(C:MF).
